# Dr. Ira Dwight Richards

**Published:** 1911-09

**Authors:** 


Dr. Ira Dwight Richards of Bergen, died August 27 after less than a week's illness. He was 82 years old and besides his wife is survived by three brothers and one sister. Dr. Richards was a pioneer in this section and was a veteran of the Civil war. Funeral on Wednesday afternoon at 2.30 o'clock.

